# Risk factors associated with multidrug-resistant tuberculosis in Espírito Santo, Brazil

**DOI:** 10.1590/S1518-8787.2017051006688

**Published:** 2017-04-18

**Authors:** Geisa Fregona, Lorrayne Belique Cosme, Cláudia Maria Marques Moreira, José Luis Bussular, Valdério do Valle Dettoni, Margareth Pretti Dalcolmo, Eliana Zandonade, Ethel Leonor Noia Maciel

**Affiliations:** I Programa de Pós-Graduação em Saúde Coletiva. Hospital Universitário Cassiano Antônio de Moraes. Universidade Federal do Espírito Santo. Vitória, ES, Brasil; II Programa de Pós-Graduação em Saúde Coletiva. Universidade Federal do Espírito Santo. Vitória, ES, Brasil; III Laboratório de Micobacteriologia da Prefeitura Municipal de Vila Velha. Vila Velha, ES, Brasil; IV Departamento de Clínica Médica. Centro de Ciências da Saúde. Universidade Federal do Espírito Santo. Vitória, ES, Brasil; V Centro de Referência Professor Hélio Fraga. Escola Nacional de Saúde Pública Sérgio Arouca. Fundação Oswaldo Cruz. Rio de Janeiro, RJ, Brasil; VI Departamento de Estatística. Universidade Federal do Espírito Santo. Vitória, ES, Brasil; VII Departamento de Enfermagem. Universidade Federal do Espírito Santo. Vitória, ES, Brasil

**Keywords:** Tuberculosis, Multidrug-Resistant, epidemiology, Drug Resistance, Multiple, Bacterial, Recurrence, Risk Factors, Socioeconomic Factors

## Abstract

**OBJECTIVE:**

To analyze the prevalence and factors associated with multidrug-resistant tuberculosis in Espírito Santo, Brazil.

**METHODS:**

This is a cross-sectional study of cases of tuberculosis tested for first-line drugs (isoniazid, rifampicin, pyrazinamide, ethambutol, and streptomycin) in Espírito Santo between 2002 and 2012. We have used laboratory data and registration of cases of tuberculosis – from the *Sistema Nacional de Agravos de Notificação* and *Sistema para Tratamentos Especiais de Tuberculose*. Individuals have been classified as resistant and non-resistant and compared in relation to the sociodemographic, clinical, and epidemiological variables. Some variables have been included in a logistic regression model to establish the factors associated with resistance.

**RESULTS:**

In the study period, 1,669 individuals underwent anti-tuberculosis drug susceptibility testing. Of these individuals, 10.6% showed resistance to any anti-tuberculosis drug. The rate of multidrug resistance observed, that is, to rifampicin and isoniazid, has been 5%. After multiple analysis, we have identified as independent factors associated with resistant tuberculosis: history of previous treatment of tuberculosis [recurrence (OR = 7.72; 95%CI 4.24–14.05) and re-entry after abandonment (OR = 3.91; 95%CI 1.81–8.43)], smoking (OR = 3.93; 95%CI 1.98–7.79), and positive culture for *Mycobacterium tuberculosis* at the time of notification of the case (OR = 3.22; 95%CI 1.15–8.99).

**CONCLUSIONS:**

The partnership between tuberculosis control programs and health teams working in the network of Primary Health Care needs to be strengthened. This would allow the identification and monitoring of individuals with a history of previous treatment of tuberculosis and smoking. Moreover, the expansion of the offer of the culture of tuberculosis and anti-tuberculosis drug susceptibility testing would provide greater diagnostic capacity for the resistant types in Espírito Santo.

## RESUMO

**OBJETIVO:**

Analisar a prevalência e fatores associados à tuberculose resistente no Espírito Santo.

**MÉTODOS:**

Estudo transversal dos casos de tuberculose testados para fármacos de primeira linha (isoniazida, rifampicina, pirazinamida, etambutol e estreptomicina) no Espírito Santo entre 2002 e 2012. Foram utilizados dados laboratoriais e de registro de casos de tuberculose – Sistema Nacional de Agravos de Notificação e Sistema para Tratamentos Especiais de Tuberculose. Os indivíduos foram classificados em resistentes e não resistentes, e comparados para variáveis sociodemográficas, clínicas e epidemiológicas. Algumas variáveis foram inclusas em um modelo de regressão logística para estabelecimento de fatores associados à resistência.

**RESULTADOS:**

No período do estudo, 1.669 indivíduos tiveram o teste de sensibilidade aos fármacos antituberculose realizado. Destes, 10,6% apresentaram resistência a qualquer droga antituberculose. A taxa de multirresistência observada, isto é, à rifampicina e isoniazida, foi de 5%. Após a análise múltipla, foram identificados como fatores associados independentes para tuberculose resistente: história de tratamento prévio para tuberculose [Recidiva (OR = 7,72; IC95% 4,24–14,05) e reingresso após abandono (OR = 3,91; IC95% 1,81–8,43)], tabagismo (OR = 3,93; IC95% 1,98–7,79) e cultura positiva para *Mycobacterium tuberculosis* no momento da notificação do caso (OR = 3,22; IC95% 1,15–8,99).

**CONCLUSÕES:**

É necessário o fortalecimento da parceria entre os programas de controle de tuberculose e as equipes de saúde que atuam na rede de Atenção Primária à Saúde. Isso possibilitaria identificar e acompanhar indivíduos com história de tratamento prévio para tuberculose e tabagismo. Além disso, a ampliação da oferta de cultura e Teste de Sensibilidade a fármacos antituberculose proporcionaria maior capacidade diagnóstica para as formas resistentes no Espírito Santo.

## INTRODUCTION

The emergence of drug-resistant strains of *Mycobacterium tuberculosis* (Mtb) is a great challenge for the elimination of tuberculosis (TB) worldwide. The World Health Organization (WHO) estimates that 3.5% of new cases and 20.5% of previously treated cases of TB are multidrug-resistant tuberculosis (MDR-TB). This is defined as *in vitro* resistance to at least two of the most potent drugs used in the conventional treatment of the disease, rifampicin (R) and isoniazid (I)^24^.

Mortality and incidence of cases of TB have decreased in Brazil over the last decade. However, Brazil remains among the 22 countries with high burden of the disease in the world and faces major difficulties related to public health systems in promoting an effective control of new cases of TB^16^. Approximately 4,064 cases of MDR-TB were reported in Brazil between 2000 and 2008. In 2014, 260 new cases of monoresistance (resistance to one anti-tuberculosis drug), 81 cases of polyresistance (resistance to two or more anti-tuberculosis drugs, except R and I), 374 cases of MDR-TB, and 56 cases of XDR-TB (cases which, in addition to resistance to R and I, present additional resistance to a fluoroquinolone and an injectable second-line drug^16^) were notified in the *Sistema de Informação de Tratamentos Especiais de Tuberculose* (SITETB – Information System of Special Treatment of Tuberculosis). Although Brazil has an undervalued number compared to other countries^24^, the problem of resistance in the country is more focused on its potential to be spread than on its magnitude^6^.

The State of Espírito Santo has presented a 13% reduction in the incidence rates of cases of TB in the last 10 years. The incidence rate was 40.6/100,000 inhabitants in 2003 and 35.2/100,000 inhabitants in 2012. The rates of cure and abandonment of treatment in the same period were, on average, 78% and 8%, respectively^16^. Most cases (60%) are concentrated in the Metropolitan Area of Greater Vitória (RMGV), formed by the capital, Vitória, and six other municipalities of great political and economic importance. Since 2002, laboratory data for anti-tuberculosis Drug Sensitivity Testing (DST) are interconnected and available to public health services. In addition, four municipalities of Greater Vitória offer universal culture for pulmonary samples, which has allowed the increase of 23.0% of the diagnosis of TB in the region^19^. However, data on resistance to Mtb are little explored^22^.

Thus, this study has aimed to describe cases of TB resistant to first-line drugs and identify factors associated with the presence of resistance in Espírito Santo.

## METHODS

This is a cross-sectional study of tuberculosis cases tested for first-line drugs (rifampicin, isoniazid, pyrazinamide, ethambutol, and streptomycin) in Espírito Santo between 2002 and 2012.

Espírito Santo is the second smallest State in Southeastern Brazil, with 46,095,583 km^2^. It has a population of approximately 3,885,049 inhabitants distributed in 78 municipalities, of which nine are classified as priority for TB control by the Ministry of Health^16^.

The cases included in this study have been bacteriologically confirmed, with positive culture for Mtb and DST performed according to the criteria established by the *Programa Nacional de Controle da Tuberculose* (PNCT/MS/Brazil – National Tuberculosis Control Program). The recommendation for DST includes: retreatment cases (recurrence and re-entry after abandonment), death during treatment, HIV-infected individuals, persons deprived of liberty or if institutionalized, and known contacts of resistant cases^a^.

The smears of samples and cultures for Mtb were, respectively, stained by the Ziehl-Neelsen method and grown on Löwenstein-Jensen (LJ) or Ogawa-Kudoh solid medium. After a maximum period of 60 days, we used a conventional biochemical method to identify the species. The DST were performed by the method of proportions or automated method (BACTEC MGIT 960/BD; Becton Dickinson, Sparks, MD, USA) at the *Laboratório Central de Saúde Pública* of Espírito Santo (LACEN-ES), for first-line drugs^a^.

We have classified as “resistant” individuals who had isolated strains of Mtb with *in vitro* resistance to at least one of the first-line drugs and as “non-resistant” individuals who did not show resistance to any of the drugs tested. We have excluded cases diagnosed as non-tuberculous mycobacteria and individuals not living in Espírito Santo.

The data on DST have been collected using software which stores laboratory data, called TB Notes. This software is present in two reference laboratories of the State to carry out culture and DST, being they the mycobacteriology laboratory of the *Núcleo de Doenças Infecciosas* of the *Universidade Federal do Espírito Santo* (NDI-UFES) and the LACEN-ES. Both are certified to perform these procedures. The LACEN-ES is under the supervision of the *Centro de Referência Professor Hélio Fraga* of Rio de Janeiro (CRPHF-RJ), a national reference for the performance of DST for first- and second-line anti-tuberculosis drugs.

For the description of the socio-demographic and clinical/epidemiological characteristics and for the analysis of risk factors for resistance, the data were researched on the main information systems (IS) that register cases of TB: the *Sistema de Informação de Agravos de Notificação* (SINAN – Information System of Reportable Diseases) and SITETB.

The sociodemographic variables used were: gender (male, female), race (white, non-white), years of study (illiterate, one to four years, five to eight years, nine to twelve years, twelve years or more), age (in years: < 20, 20 to 39, 40 to 59, 60 or more), and institutionalization (yes, no). Regarding health history, we analyzed the presence/absence of HIV infection, alcoholism, diabetes mellitus, mental illness, smoking, and use of illicit drugs.

We selected for the analysis of characteristics of the disease and its treatment the variables: type of case (new case, recurrence, re-entry after abandonment, transfer), number of prior treatments of TB (none, one, two, three or more), radiological examination of the thorax (suspected, normal), tuberculin test (no reaction, weak reaction, strong reaction), clinical type (pulmonary, extrapulmonary, pulmonary + extrapulmonary), sputum smear microscopy in the diagnosis (positive, negative), sputum culture in the diagnosis (positive, negative), other material culture in the diagnosis (positive, negative), and ending (cure, abandonment, death by TB, death by other causes, transfer, MDR-TB). We verified the existence of more than one record of treatment for the same individual in the study period to ensure the reliability of the information about history of prior treatment of TB and number of treatments carried out.

The rates of resistance (to any drug and MDR-TB) were expressed as the proportion of resistant individuals among those tested. We used Pearson’s Chi-square test for the categorical variables and t-test for the numeric variables to observe statistical differences between the groups of cases of resistant and non-resistant TB. The multiple logistic regression analysis was used to calculate the adjusted odds ratios (OR). We considered 5% significance for the input of variables in the model and the method ENTER to choose the variables (which considers all variables included in the model). The significance level adopted in all analyses was 5%.

We created a database as a spreadsheet in Microsoft Office Excel^®^, and we summarized the data in the statistical program SPSS, version 18 (Chicago, IL, USA).

This study has been approved by the research ethics committee of the *Centro de Ciências da Saúde* of the *Universidade Federal do Espírito Santo* (Process 201.111/2013).

## RESULTS

Approximately 15,851 cases of tuberculosis were reported in Espírito Santo between 2002 and 2012. We have found 1,669 individuals in the TB Notes with DST performed in the same period. Of these individuals, 89% had no *in vitro* resistance to first-line drugs, and 10.6% presented some type of resistance (Figure).

**Figure:**
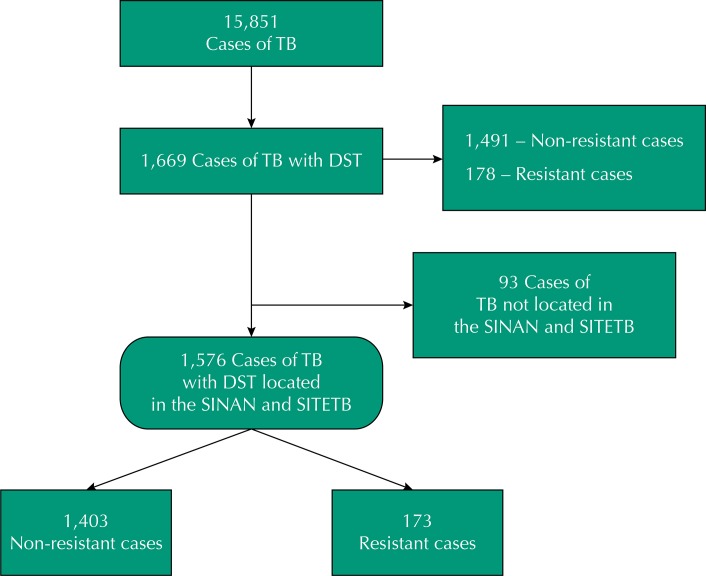
Flowchart of TB cases notified. State of Espírito Santo, Brazil, 2002 to 2012.

TB: tuberculosis; DST: anti-tuberculosis drug susceptibility testing; SINAN: Information System of Reportable Diseases; SITETB: Information System for Special Treatment of Tuberculosis

The monoresistance to isoniazid followed by streptomycin were the most common (2.1% and 0.9% of the cases tested, respectively). The rate of multidrug resistance was 5%, and most of them showed resistance to isoniazid + rifampicin (2.3%) (Table 1). Of the individuals who underwent DST, 93 were not located in the IS for the treatment of tuberculosis, and of these three were classified as resistant. Therefore, we have collected the sociodemographic, clinical, and epidemiological information of 1,576 individuals (Figure).

**Table 1:** Profile of first-line drug resistance. State of Espírito Santo, Brazil, 2002 to 2012.

Variable	n	%
Profile of resistance		
Total strains tested	1,669	100
Non-resistant	1,491	89.3
Any resistance	178	10.7
H	139	8.3
R	95	5.7
Z	48	2.9
E	22	1.3
S	58	3.5
Monoresistance (total)	67	4.0
H	36	2.1
R	6	0.4
Z	2	0.1
E	7	0.4
S	16	0.9
Polyresistance (total)	26	1.5
H+Z	1	0.1
H+E	5	0.3
H+S	8	0.5
R+Z	3	0.2
Z+S	3	0.2
E+S	1	0.1
H+Z+S	3	0.2
H+E+S	1	0.1
R+E+S	1	0.1
MDR-TB (total)	85	5.1
RH	38	2.3
RH+Z	17	1.0
RH+E	2	0.1
RH+S	9	0.5
RH+Z+E	3	0.2
RH+Z+S	14	0.8
RH+Z+E+S	2	0.1

H: isoniazid; R: rifampicin; Z: pyrazinamide; E: ethambutol; S: streptomycin; Any resistance: resistance to any anti-tuberculosis drug; MDR-TB: Multidrug-resistant Tuberculosis; RH: Rifampicin + Isoniazid

Most individuals (84%) were from the nine priority municipalities for TB control, and 67% were from the four municipalities that have universal culture for pulmonary samples (Cariacica, Serra, Vila Velha, and Vitória). In the comparison between the resistant and non-resistant groups, no statistically significant differences were found for the sociodemographic variables. There was a predominance of males, non-whites, and low education level – most (39%) had complete elementary school (Table 2). Average age (in years) in the non-resistant group was 36.8 (SD = 14.5) and in the resistant group it was 37.6 (SD = 13.5; p = 0.482).

**Table 2:** Distribution of the socio-demographic characteristics of individuals diagnosed with TB who underwent DST. State of Espírito Santo, Brazil, 2002 to 2012.

Characteristic	Total		Groups				p*

			Non-resistant		Resistant		
		
	n	%	n	%	n	%	
Gender	1,576						
Male		71.0	991	71.0	132	76.0	0.120
Female		29.0	412	29.0	41	24.0	
Race	1,428						
White		29.0	362	29.0	55	33.0	0.236
Non-white		71.0	900	71.0	111	67.0	
Education level (years)	1,145						
Illiterate		8.0	79	8.0	9	7.0	0.749
1 to 4		28.0	282	28.0	37	29.0	
5 to 8		39.0	400	39.0	44	34.0	
9 to 12		21.0	209	21.0	32	25.0	
≥ 12		5.0	47	5.0	6	5.0	
Age (years)	1,557						
< 20		8.0	105	8.0	14	8.0	0.258
20-39		52.0	734	53.0	81	48.0	
40-59		33.0	448	32.0	65	39.0	
≥ 60		7.0	102	7.0	8	5.0	
Institutionalization	881						
No		89.0	680	89.0	106	93.0	0.165
Yes		11.0	87	11.0	8	7.0	
HIV	1,193						
Positive		11.0	112	11.0	14	10.0	0.842
Negative		89.0	942	89.0	125	90.0	
Alcohol	909						
Yes		35.0	267	34.0	54	45.0	0.021
No		65.0	521	66.0	67	55.0	
Diabetes	769						
Yes		8.0	54	8.0	11	10.0	0.529
No		92.0	605	92.0	99	90.0	
Mental illness	815						
Yes		2.0	17	2.0	3	3.0	0.908
No		98.0	683	98.0	112	97.0	
Smoking	609						
Yes		13.0	48	9.0	32	34.0	< 0.001
No		87.0	467	91.0	62	66.0	
Use of drugs	604						
Yes		12.0	65	12.0	9	13.0	0.908
No		88.0	468	88.0	62	87.0	

TB: tuberculosis; DST: anti-tuberculosis drug susceptibility testing

* Pearson’s Chi-square test.

There was no difference in the distribution of cases of HIV infection between the groups. Statistical differences were observed for the variables alcohol consumption (p = 0.021) and smoking (p < 0.001), both more common among the resistant than the non-resistant individuals (45% *versus* 34% and 34% *versus* 9%, respectively) (Table 2).

Statistical differences were observed for the clinical and epidemiological variables. For the variable type of case (p < 0.001), the non-resistant group showed the highest percentage of new cases of TB compared to the resistant group (77% *versus* 45%). On the other hand, the number of cases of retreatment was higher in the resistant group (53% *versus* 21%).

The registration of one previous episode of TB was more common among resistant individuals than among the non-resistant individuals (30% *versus* 11%). The positive culture at the time of diagnosis (p = 0.023) was more frequent in the resistant group than in the non-resistant group, 95% *versus* 85%. The proportion of cured cases among the non-resistant group was greater than among the resistant group (83% *versus* 77%) (Table 3).

**Table 3:** Distribution of the clinical and epidemiological characteristics of individuals diagnosed with TB who underwent DST. State of Espírito Santo, Brazil, 2002 to 2012.

Characteristic	Total		Groups				p^a^

			Non-resistant		Resistant		
		
	n	%	n	%	n	%	
Type of case	1,575						
New case		74.0	1,083	77.0	77	45.0	< 0.001
Recurrence		14.0	155	11.0	70	40.0	
Re-entry after abandonment		10.0	136	10.0	23	13.0	
Transfer		2.0	28	2.0	3	2.0	
Number of previous treatments	1,576						
No treatment		80.0	1,183	84.0	80	46.0	< 0.001
1		13.0	150	11.0	52	30.0	
2		6.0	59	4.0	29	17.0	
≥ 3		1.0	11	1.0	12	7.0	
Chest x-ray	1,487						
Suspected		98.0	1,298	98.0	161	98.0	0.587
Normal		2.0	24	2.0	4	2.0	
Tuberculin test	384						
No reaction		18	65	19	3	7	0.122
Weak reaction		7	22	6	4	9	
Strong reaction		76	253	74	37	84	
Type	1,575						
Pulmonary		94	1323	94	162	94	0.916
Extrapulmonary		2	34	2	5	3	
Pulmonary + Extrapulmonary		3	45	3	6	3	
Sputum smear microscopy	1,514						
Positive		83	1,121	83	143	85	0.675
Negative		17	224	17	26	15	
Sputum culture	1,124						
Positive		89	863	88	138	95	0.023
Negative		11	115	12	8	5	
Other material culture	127						
Positive	87	69	74	69	13	65	0.713
Negative	40	31	33	31	7	35	
Ending	1,571						
Cure		82	1,156	83	131	77	< 0.001
Abandonment		8	108	8	11	6	
Death by TB		2	28	2	5	3	
Death by other causes		3	40	3	6	4	
Transfer		4	59	4	6	4	
MDR-TB^b^		1	3	0	11	6	

TB: tuberculosis; MDR-TB: multidrug-resistant tuberculosis; DST: anti-tuberculosis drug susceptibility testing

^a^ Pearson’s Chi-square Test.

^b^ Data available in the *Sistema de Informação de Agravos de Notificação* (SINAN – Information System of Reportable Diseases) from 2007.

Type of case, smoking, and sputum culture at the time of diagnosis were important associated factors for resistance to anti-tuberculosis drugs after the analysis adjusted for variables included in the logistic regression model (Table 4).

**Table 4:** Multiple analysis of the clinical and epidemiological characteristics associated with resistance in individuals with tuberculosis who underwent DST. State of Espírito Santo, Brazil, 2002 to 2012.

Characteristic	Crude OR			Adjusted OR		
	
	p	OR	95%CI	p	OR	95%CI
Type of treatment						
New case		1			1	
Recurrence	< 0.001	6.35	4.41–9.15	< 0.001	7.72	4.24–14.05
Re-entry after abandonment	0.001	2.38	1.44–3.92	0.001	3.91	1.81–8.43
Transfer	0.508	1.51	0.45–5.07	0.999		
Number of previous treatments*						
No treatment		1				
1	< 0.001	5.13	3.48–7.56			
2	< 0.001	7.27	4.41–11.97			
≥ 3	< 0.001	16.13	6.90–37.70			
Alcohol						
Yes	0.022	1.57	1.07–2.32	0.933	1.03	0.55–1.92
No		1			1	
Smoking						
Yes	< 0.001	5.02	2.99–8.45	< 0.001	3.93	1.98–7.79
No		1			1	
Sputum culture						
Positive	0.027	2.30	1.10–4.81	0.026	3.22	1.15–8.99
Negative		1			1	

DST: anti-tuberculosis drug susceptibility testing

* Variable is not part of the logistic regression model by collinearity.

N = 428 (27.2%)

Individuals with recurrence had adjusted odds ratio (OR) of 7.72 (p < 0.001; 95%CI 4.24–14.05) for resistance to anti-tuberculosis drugs in relation to individuals who had no history of previous treatment (new cases). For cases of re-entry after abandonment, adjusted OR was 3.91 (p < 0.001; 95%CI 1.81–8.43) also compared to new cases.

Individuals with a history of smoking presented adjusted OR of 3.93 (p < 0.001; 95%CI 1.98–7.79) for resistance, using as reference non-smokers. The positive sputum culture for Mtb presented weaker association with outcome (resistance), and adjusted OR of 3.22 (p = 0.026; 95%CI 1.15–8.99). Number of previous treatments, despite significant (p < 0.001), did not become part of the logistic regression model as it presents collinearity with the variable type of case.

## DISCUSSION

We have found strong association between the number of previous treatments of TB, smoking, and positive culture at the time of diagnosis with cases of resistant TB. There is a consensus in the literature that previous treatment of TB is a strong risk factor for bacterial resistance to anti-tuberculosis drugs^8^ ^,^ ^9^ ^,^ ^11^ ^,^ ^17^. Our results have shown that cases of recurrence have twice the odds ratio for the occurrence of resistance in relation to retreatment by re-entry after abandonment. This can be explained by the increased contact with anti-tuberculosis drugs. A study conducted in eleven countries has shown that the longer the time of exposure to anti-tuberculosis drugs, the greater the chance of occurrence of resistance^8^.

Another risk factor presented in this study was the habit of smoking. Despite increasing the risk of active TB^12^ ^,^ ^20^, we have found no sufficient evidence in the literature that smoking is an important risk factor for resistance^7^. However, studies show smoking as a factor associated with the failure of treatment of TB. The time to sputum culture conversion from positive to negative among smokers is greater than among nonsmokers after the second month of the start of treatment with both first-line^13^ ^,^ ^23^ and second-line drugs^1^ ^,^ ^14^.

There is a strong association between smoking and recurrence of TB. A consistent study has shown that smokers are 2.5 times more likely to have a recurrence than nonsmokers^2^. Thus, the relationship between smoking, previous treatment of TB (recurrence), and resistance to anti-tuberculosis drugs deserve to be better investigated.

The occurrence of one previous treatment was more frequent among previously treated individuals. The presence of universal culture covering areas of high population concentration in Espírito Santo may have contributed to the earlier suspicion and confirmation of resistant cases^22^. These data are probably related to the greater number of positive cultures at diagnosis among resistant cases. The recent seizure of a new technology based on a rapid molecular testing for TB (RMT-TB), as an important tool in detecting the resistance to R for new cases of TB, does not exclude the culture and DST by conventional methods in Brazil^16^. Universal culture is a recommendation of the WHO, although it is not yet a reality in many places in Brazil and in the world^24^.

Among the resistant cases, 45% had never been treated for TB. This may suggest the evidence of active transmission in the population, as we also have not found association with other variables commonly reported in the literature that may point to groups at higher risk for resistant TB, such as: age, gender, HIV infection, and history of institutionalization^8^ ^,^ ^18^. Similar result has been described in an European study, which has assessed cases of MDR-TB in sixteen countries, showing that 52.4% of them had never received anti-tuberculosis treatment (59.2%, 74.4%, and 38.7% in countries with low, intermediate, and high incidence of TB, respectively)^11^. This can be explained by migratory movements of individuals in search of better living conditions. Espírito Santo is the main route linking the Northeast and Southeast regions of the country and is located between two large States that have high incidence rates of TB, Rio de Janeiro and Bahia^16^.

For this hypothesis, we must consider the possibility of underreporting of cases. This study is based on secondary data and no interview or recent contact was performed with the patients reported in the period of study to investigate the history of treatment of TB.

The rate of resistance to any drug was 10.6% and for MDR-TB it was 5%. Among the Brazilian studies with secondary data, there is great variability of results, given the distinct characteristics of the populations studied. In these studies, the rate of resistance to any drug has varied from 9.4% to 19.2% and for MDR-TB it has varied from 3.4% to 15%^4^ ^,^ ^5^ ^,^ ^10^ ^,^ ^15^ ^,^ ^21^.

Data from the first national resistance survey conducted between 1995 and 1997, carried out prospectively, presented the same rate of resistance to any drug of 10.6%. However, the rate of multidrug resistance was 2.2%, half of that observed in this study^3^. A retrospective study in Israel has found results very close to those observed herein. The rate of resistance to any drug was 12.5% and for MDR-TB it was 5.8%^18^.

There was a predominance of isolated resistance to isoniazid followed by streptomycin. For the cases of MDR-TB, the highest frequency was observed for isoniazid + rifampicin. Other Brazilian studies have found a similar situation^3^ ^-^ ^5^ ^,^ ^15^ ^,^ ^21^. We believe that this result is a reflection of a consolidated policy of notification and treatment of cases of TB made available free of charge and offered exclusively by public health services. On the other hand, perhaps there is a different profile of resistance in regions where there has not been an effective policy of control and treatment of cases^1^ ^,^ ^6^.

Of the 1,669 cases of TB with DST, 5.6% were not located in the IS. We have not considered it a statistically significant loss for the final analyses, as we have obtained 94.4% of the data. However, this fact raises some possible explanations. The first of them is that these cases may have been diagnosed in Espírito Santo, but they were sent to other States for treatment. Another possibility would be of these individuals being outside the study period and not identified in the IS, or yet, they could have been lost in the public health system without access to appropriate treatment, characterizing primary abandonment of treatment.

Espírito Santo, following the rules of the PNCT, does not perform DST in all new cases of TB^a^. Therefore, the rates of resistance presented here may be underestimated. The missing information in the databases has been classified as missing in the bivariate analyses. For the logistic regression, we have used 30% of the available data. However, we consider the number of 428 individuals as enough for the final analysis.

The results presented in this study show that the partnership between Tb control programs (PCT) and health teams working in Primary Health Care needs to be strengthened. Individuals with a history of previous treatment of TB and smoking should be identified and monitored. This can contribute to reduce the adverse outcomes in the treatment of TB. Another important point is the orientation to stop smoking among individuals under treatment of TB. In addition to other health benefits, it can prevent the emergence of cases of resistant TB.

It is essential to expand the provision of culture and DST, providing greater diagnostic capacity for the types of resistant TB in Espírito Santo. The early diagnosis and treatment of these cases prevent the circulation of strains of Mtb, reducing the number of primary cases, i.e., individuals who have never been treated for TB and who get sick with resistant strains.

## Footnotes

a Ministério da Saúde (BR), Secretaria de Vigilância em Saúde, Departamento de Vigilância Epidemiológica. Manual nacional de vigilância laboratorial da tuberculose e outras micobactérias. Brasília (DF); 2008 [cited 2016 Sep 20]. Available from: http://bvsms.saude.gov.br/bvs/publicacoes/manual_vigilancia_laboratorial_tuberculose.pdf
